# Safety and efficacy of long-term esomeprazole 20 mg in Japanese patients with a history of peptic ulcer receiving daily non-steroidal anti-inflammatory drugs

**DOI:** 10.1186/1471-230X-13-54

**Published:** 2013-03-26

**Authors:** Kentaro Sugano, Yoshikazu Kinoshita, Hiroto Miwa, Tsutomu Takeuchi

**Affiliations:** 1 Department of Medicine, Jichi Medical University, 3311-1 Yakushiji, Shimotsuke, Tochigi, 329-0498, Japan; 2 Department of Gastroenterology and Hepatology, Shimane University School of Medicine, Shimane, Japan; 3 Department of Internal Medicine, Hyogo College of Medicine, Hyogo, Japan; 4 Department of Internal Medicine, Keio University School of Medicine, Tokyo, Japan

**Keywords:** Esomeprazole, Non-steroidal anti-inflammatory drugs, Peptic ulcer, Safety

## Abstract

**Background:**

Non-steroidal anti-inflammatory drugs (NSAIDs) are an effective and common treatment for chronic pain disorders, but long-term use is associated with risk of potentially life-threatening gastrointestinal adverse events (AEs). The proton pump inhibitor esomeprazole has been found to be effective for gastroprotection in NSAID users, but few long-term studies have been conducted in Japan.

**Methods:**

This was an open-label, multicentre, single-arm, prospective 1-year study of treatment with esomeprazole (20 mg once daily) in Japanese patients (aged ≥20 years) with endoscopic evidence of previous peptic ulcer and receiving daily oral NSAID therapy (at a stable dose) for a chronic condition. Eligibility was not dictated by type of oral NSAID. The primary objective was to determine long-term safety and tolerability of esomeprazole. Efficacy for prevention of peptic ulcers was also determined (Kaplan-Meier method). All statistical analyses were descriptive.

**Results:**

A total of 130 patients (73.1% women, mean age 62.1 years, 43.8% *Helicobacter pylori*-positive) received treatment with esomeprazole in addition to long-term NSAID therapy (most commonly for rheumatoid arthritis [n=42] and osteoarthritis [n=34]). Loxoprofen, meloxicam and diclofenac were the most commonly used NSAIDs; cyclo-oxygenase (COX)-2 selective agents were used by 16.2% of patients (n=21). Long-term compliance with esomeprazole (capsule counts) was >75% for the majority of patients. Although 16.9% of patients (n=22) experienced AEs judged to be possibly related to treatment with esomeprazole, they were mostly mild and transient. The most commonly reported possibly treatment-related AEs were abnormal hepatic function, headache, increased γ-glutamyltransferase levels and muscle spasms (2 patients each). Overall, 95.9% (95% confidence interval: 92.3, 99.4) of patients remained ulcer free at 1 year.

**Conclusion:**

Long-term treatment with esomeprazole (20 mg once daily) is well tolerated and efficacious for preventing ulcer recurrence in Japanese NSAID users with a history of peptic ulcer.

**Trial registration:**

ClinicalTrials.gov identifier NCT00595517.

## Background

Chronic pain conditions, such as osteoarthritis and rheumatoid arthritis, are common in Japan. For example, the prevalence of gonarthrosis or gonarthritis among women in Japan is 36–63%, depending on age [1], and some 320,000 Japanese individuals are affected by rheumatoid arthritis according to a 2002 survey [2]. Non-steroidal anti-inflammatory drugs (NSAIDs) provide effective pain management for patients with such conditions [3], and are widely prescribed in Japan and other Asian countries [4,5].

While effective for relief of pain, the long-term use of NSAIDs may cause gastrointestinal (GI) adverse events (AEs) ranging from mild upper GI symptoms such as dyspepsia [6] to peptic ulcers, which can lead to life-threatening complications if the ulcers perforate or haemorrhage [6,7]. A pooled analysis of 21 randomised, parallel-group studies of celecoxib and non-selective NSAIDs, for example, identified GI symptoms (abdominal pain, dyspepsia, nausea, diarrhoea and flatulence) in 16% of patients taking celecoxib and around 20–24% of those taking diclofenac, ibuprofen or naproxen [6]. With regard to the risk of peptic ulcers, a study of patients with osteoarthritis found that up to 17% of patients developed such lesions within 12 weeks of commencing treatment with non-selective NSAIDs such as ibuprofen and diclofenac [8]. This compares with a background incidence of 3.3% in a general population, based on a meta-analysis of the placebo treatment arms of 36 clinical trials of NSAIDs [9]. Risk factors for upper GI events among NSAID users are numerous, and include older age, a history of previous GI events (e.g. bleeds) and use of higher NSAID doses [10,11].

Minimising the risk of potentially serious GI events in long-term NSAID users is clearly appropriate, especially for those patients at increased risk. The proton pump inhibitor (PPI) esomeprazole has been shown to be efficacious in the prevention of peptic ulcers and upper GI symptoms related to NSAID use in European, North American and Japanese patients [12-14], but few long-term studies have been conducted in the corresponding Japanese population. The present study therefore investigated the safety and tolerability of long-term treatment with esomeprazole 20 mg in Japanese patients with an endoscopically-confirmed history of peptic ulcer who were also receiving daily NSAID therapy. Efficacy of esomeprazole for prevention of peptic ulcers and gastrointestinal symptoms was also determined.

## Methods

### Study design

This was an open-label, multicentre, single-arm, prospective study in Japanese patients with a history of peptic ulcer receiving daily NSAID therapy (ClinicalTrials.gov identifier: NCT00595517; study code: D961HC00005). The study was conducted in accordance with the ethical principles of the Declaration of Helsinki, ICH/Good Clinical Practice and good clinical practice regulatory requirements in Japan [15]. The study protocol was approved by the Institutional Review Board of Shirakawa Hospital, Fukushima and approved by an independent institutional review board or research ethics committee at each participating centre. Written informed consent was a requirement for patients to enter the study, which was conducted between October 2007 and September 2009.

### Patients and treatment

Japanese men and women aged ≥20 years with a history of peptic ulcer were recruited to the study. History of peptic ulcer was defined as evidence of ulcer scarring during endoscopy performed within 2 weeks of enrolment. Scarring was evaluated according to the Sakita/Miwa classification [16], i.e. ‘red scar’ (S1): hyperaemia remains in the centre of the scar; ‘white scar’ (S2): hyperaemia in the scar has disappeared, turning into the same colour as adjacent mucosa. Patients were also required to have a chronic condition (e.g. rheumatoid arthritis, osteoarthritis) that necessitated daily oral NSAID treatment (≥5 days/week) at a stable dose for the duration of the study. Eligibility was not dictated by type of oral NSAID. Use of additional oral/topical NSAIDs was permitted to maintain patient wellbeing. Exclusion criteria included severe liver or renal disease, abnormal liver or kidney function tests, a history of malignant disease or other defined conditions including Barrett’s esophagus, esophagitis, pancreatitis, uncontrolled diabetes mellitus or severe cardiovascular or cerebrovascular disease. Presence of active ulcer was not permitted, nor was continuous use of pre-specified concomitant medications including anticoagulant/antiplatelet therapy (including aspirin <325 mg/day), PPIs, mucosal protectants, H_2_-receptor antagonists, antacids or prostaglandin analogues indicated for peptic ulcer. In accordance with the study protocol, treatment with *Helicobacter pylori*-eradication therapy was also not permitted after enrolment to avoid the potential confounding effect on ulcer recurrent rates. Women of child-bearing potential had to have a negative pregnancy test at the screening visit, and to use effective contraception during the study.

Patients received one capsule of esomeprazole 20 mg orally once daily (od), after breakfast, for 52 weeks in addition to physician-prescribed NSAID therapy. Compliance with esomeprazole treatment was determined by checking the number of remaining capsules returned at regular clinic visits (every 4–8 weeks). Subjects were also asked to record their daily NSAID use in a diary, and study investigators checked the NSAID treatment diary at each clinic visit.

### Outcomes

The primary objective was the assessment of safety and tolerability of esomeprazole 20 mg od during 52 weeks of continuous treatment, as determined by AE reports, clinical laboratory tests (haematology, biochemistry and urinalysis) and measurement of vital signs at each clinic visit. Information on AEs (spontaneously reported, and in response to open questioning) was collected according to standard regulatory requirements, an AE being defined as the development of an undesirable medical condition (or the deterioration of a pre-existing medical condition) following or during exposure to study medication. In this regard, an undesirable medical condition was considered to be symptoms, signs or the abnormal results of any investigation. AEs were evaluated by investigators in terms of seriousness, maximum intensity (mild, moderate or severe), outcome and possible causality with study medication. A genetic test was also completed for each patient, in order to determine cytochrome P450 (CYP) 2C19 genotype; based on the result of this test, patients were classified as ‘extensive metabolisers’ (homo- and hetero- types) or ‘poor metabolisers’. The latter subgroup was defined as those with *2 and *3 variant alleles. Such tests were completed by a central laboratory (Mitsubishi Chemical Medience Corporation, Tokyo, Japan).

The secondary objective was to assess the efficacy of esomeprazole 20 mg od by the following evaluations:

•Endoscopic evidence of peptic ulcer and severity of gastric mucosal lesions (evaluated by modified LANZA score [17], if present) at weeks 4, 12, 24 and 52.

•Presence and severity (mild, moderate or severe) of investigator-assessed pre-specified dyspeptic symptoms every 4 weeks to week 52.

With regard to the assessment of gastric mucosal lesions, patients were assigned a modified LANZA score as follows: absence of haemorrhage and erosion (0); one haemorrhage or erosion (+1); 2–10 haemorrhages or erosions (+2); 11–25 haemorrhages or erosions (+3); and >25 haemorrhages, erosions or an ulcer (+4). In terms of the symptom assessment, patients were asked to report on the presence and severity of specific symptoms (epigastric pain or discomfort, abdominal fullness, nausea/vomiting, heartburn and anorexia) during the 7 days before clinic visits.

### Statistical analyses

All analyses were descriptive. The safety evaluation (primary objective) comprised all patients who had taken at least one dose of study medication and from whom any post-dose data were available (safety analysis set). Efficacy outcomes (secondary objective) were analysed for the full analysis set (FAS), which consisted of patients who took at least one dose of study medication and who had no active peptic ulcer at baseline. Peptic ulcer-free rates (and 95% confidence intervals [CIs]) were estimated using the Kaplan-Meier method. For the purposes of this analysis, an event was defined as endoscopy-confirmed peptic ulcer; time-to-event comprised the time between the date of initial administration and the date of event occurrence. The last endoscopy test day was the data cut-off for patients who did not develop ulcer(s) throughout the study period (the last-observation-carried-forward approach was not used). Observed ulcer-free rates were also recorded according to endoscopic evaluation, and additional analyses were completed to evaluate the effect of risk factors on ulcer-free rates. Supplementary efficacy analyses were completed according to an independent review of each patient’s endoscopic images by a Central Evaluation Committee (Y.K. and H.M.). The change of modified LANZA score and symptoms was evaluated using shift tables.

In view of the study design and duration, a sample size of over 100 completing patients was deemed sufficient to meet the primary objectives of the study.

## Results

### Patients

A total of 395 patients were screened for inclusion, of whom 265 patients were not registered for the study because of non-eligibility (n=247) or voluntary discontinuation by patients (n=18). Almost all of the non-eligible patients had no evidence of ulcer scarring during endoscopic assessment, and thereby did not meet the study inclusion criterion (history of peptic ulcer). A total of 130 patients therefore entered the study and received treatment with esomeprazole. Enrolled patients were mainly women (73.1%), and the mean age was 62.1 years (Table 1). More than half (51.5%) of the patients were aged 65 years or older, and 43.8% were *H. pylor*i-positive (serology test). The most frequent diagnoses that necessitated long-term NSAID therapy were rheumatoid arthritis (n=42, 32.3%) and osteoarthritis (n=34, 26.2%); the remaining patients had other chronic conditions (mostly lumbago and cervical spondylitis; n=54, 41.5%). Loxoprofen, meloxicam and diclofenac were the most commonly used NSAIDs at baseline (40.0%, 17.7% and 11.5%, respectively). COX-2 selective NSAIDs (celecoxib, etodolac or nabumetone) were used by 16.2% of patients (n=21). Overall, few patients were using two or more NSAIDs at baseline (n=5, 3.8%). Some 24.6% of patients (n=32) were receiving concomitant treatment with corticosteroids, most commonly prednisolone. A total of 22 patients (16.9%) were CYP2C19 poor metabolisers.

**Table 1:** Baseline characteristics of patients (full analysis set)

**Variable**	**Esomeprazole 20 mg once daily (n=130)**
Women	95 (73.1)
Age	
Mean±SD (range), years	62.1±12.6 (32–84)
≤64 years	63 (48.5)
≥65 to ≤74 years	43 (33.1)
≥75 years	24 (18.5)
Type of arthritic disease	
Rheumatoid arthritis	42 (32.3)
Osteoarthritis	34 (26.2)
Other chronic condition	54 (41.5)
Duration of disease, mean±SD (range), years	6.0±6.9 (0–38)
*Helicobacter pylori* infection (serology test)	57 (43.8)
Use of corticosteroids	32 (24.6)
CYP2C19 genotype	
Poor metaboliser	22 (16.9)
Hetero extensive metaboliser	68 (52.3)
Homo extensive metaboliser	40 (30.8)

Values are presented as number of patients (%), unless otherwise stated.

**Abbreviation:** CYP2C19, cytochrome P450 2C19.

Compliance with daily esomeprazole and NSAID therapy during the course of the study was good (99% of patients took more than 75% [esomeprazole] or 70% [NSAIDs] of prescribed medication as instructed).

**Figure 1:**
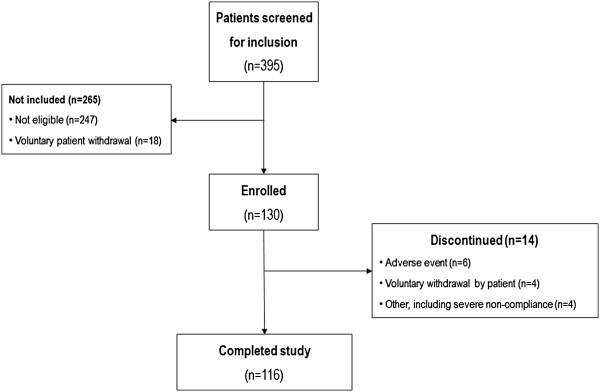
Patient flow.

A total of 116 patients completed the study (including 5 patients with recurrent ulcer). The main reasons for study discontinuation were adverse events (n=6) and voluntary patient withdrawal (n=4). A summary of patient flow through the study is shown in Figure 1.

### Primary objective: safety and tolerability

Overall, 123 patients (94.6%) reported at total of 564 AEs during the 52-week treatment period. No time-related trends were generally apparent, and the majority of AEs were of mild-to-moderate intensity and non-serious. The most commonly reported AEs were nasopharyngitis, aggravation of underlying rheumatoid arthritis and upper abdominal pain. Six patients (4.6%) discontinued from the study because of AEs, including one patient with an event (erosive gastritis) that was considered related to the study treatment (Table 2).

**Table 2:** Number of patients (%) with at least one adverse event (AE), by time interval (safety analysis set)

	**Esomeprazole 20 mg once daily**		
	**0–24 weeks (n=130)**	**24–52 weeks (n=116)**	**0–52 weeks (n=130)**
Any AE^a^	105 (80.8)	93 (80.2)	123 (94.6)
Nasopharyngitis	26 (20.0)	22 (19.0)	38 (29.2)
Rheumatoid arthritis aggravated	13 (10.0)	4 (3.4)	17 (13.1)
Upper abdominal pain	8 (6.2)	8 (6.9)	14 (10.8)
Constipation	7 (5.4)	5 (4.3)	12 (9.2)
Diarrhoea	6 (4.6)	5 (4.3)	11 (8.5)
Stomach discomfort	6 (4.6)	4 (3.4)	10 (7.7)
Osteoarthritis	4 (3.1)	5 (4.3)	9 (6.9)
Headache	6 (4.6)	2 (1.7)	8 (6.2)
Nausea	0	8 (6.9)	8 (6.2)
Anorexia	3 (2.3)	4 (3.4)	7 (5.4)
Arthralgia	3 (2.3)	4 (3.4)	7 (5.4)
Contusion	4 (3.1)	4 (3.4)	7 (5.4)
Hypertension	4 (3.1)	3 (2.6)	7 (5.4)
Stomatitis	2 (1.5)	5 (4.3)	7 (5.4)
Vomiting	3 (2.3)	4 (3.4)	7 (5.4)
Severe AEs	2 (1.5)	1 (0.9)	3 (2.3)
Non-fatal serious AEs	13 (10.0)	5 (3.8)	18 (13.8)
AEs leading to treatment discontinuation^b^	5 (3.8)	1 (0.9)	6 (4.6)

^a^Only events reported for >5% of patients are listed. Information on AEs was collected according to standard regulatory requirements, an AE being defined as the development of an undesirable medical condition (or the deterioration of a pre-existing medical condition) following or during exposure to study medication. In this regard, an undesirable medical condition was considered to be symptoms, signs or the abnormal results of any investigation.

^b^Including erosive gastritis (n=2), cerebral infarction (n=1), sore throat (n=1), lung tumour (n=1) and influenza A virus infection (n=1).

AEs possibly related to treatment, as judged by the investigator, were experienced by 16.9% of patients (n=22). These were mostly of mild intensity, transient and only single occurrences. The most commonly reported possibly treatment-related AEs were abnormal hepatic function, headache, increased γ-glutamyltransferase levels and muscle spasms (2 patients each) (Table 3).

**Table 3:** Number of patients (%) with at least one possibly treatment-related adverse event (AE) (safety analysis set)

	**Esomeprazole 20 mg once daily (n=130)**
Possibly treatment-related AEs^a^	22 (16.9)
Abnormal hepatic function	2 (1.5)
Headache	2 (1.5)
Increased GGT levels	2 (1.5)
Muscle spasms	2 (1.5)
Anorexia	1 (0.8)
Asthma	1 (0.8)
Benign skin neoplasm	1 (0.8)
Constipation	1 (0.8)
Decreased neutrophil count	1 (0.8)
Decreased platelet count	1 (0.8)
Dysgeusia	1 (0.8)
Dysphagia	1 (0.8)
Erosive gastritis	1 (0.8)
Erysipelas	1 (0.8)
Esophageal candidiasis	1 (0.8)
Gastric polyps (fundic gland polyp)	1 (0.8)
Hypertension	1 (0.8)
Increased ALT levels	1 (0.8)
Increased blood CPK levels	1 (0.8)
Increased blood urea	1 (0.8)
Nummular eczema	1 (0.8)
Pneumonia	1 (0.8)
Pruritus	1 (0.8)
Stomach discomfort	1 (0.8)
Stomatitis	1 (0.8)
Upper abdominal pain	1 (0.8)

^a^Information on AEs was collected according to standard regulatory requirements, an AE being defined as the development of an undesirable medical condition (or the deterioration of a pre-existing medical condition) following or during exposure to study medication. In this regard, an undesirable medical condition was considered to be symptoms, signs or the abnormal results of any investigation.

**Abbreviations:** ALT, alanine aminotransferase; CPK, creatine phosphokinase; GGT, γ-glutamyltransferase.

There were no deaths and no clinically important changes in laboratory test variables or vital signs consistent with safety concern during the 52-week study.

### Secondary objective: efficacy

**Figure 2:**
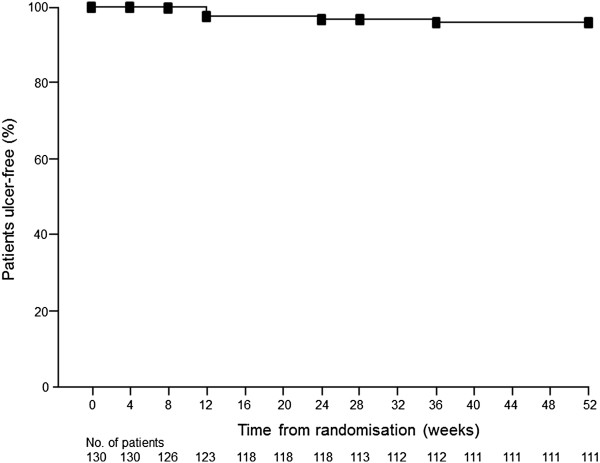
Kaplan-Meier estimated rate of sustained ulcer-free status over 1 year (full analysis set).

Overall, the majority of FAS patients remained free of peptic ulcers during the study; the estimated ulcer-free rate at 1 year was 95.9% (95% CI 92.3, 99.4; Figure 2). Observed ulcer-free rates at weeks 4, 12, 24 and 52 were 100% (130/130), 97.7% (127/130), 96.9% (126/130) and 96.2% (125/130), respectively. All 5 patients (4 women and 1 man) who did not remain ulcer-free developed gastric ulcers. Clinical characteristic of these cases included one patient with concomitant use of corticosteroids and another patient with confirmed *H. pylori* infection (serology test). Ulcer-free rates at 1 year, by demographic or other patient characteristics at baseline, are shown in Table 4. Similar results were apparent for the efficacy analysis of the Central Evaluation Committee (data not shown).

**Table 4:** Kaplan-Meier estimated sustained ulcer-free rates (95% confidence interval) at 52 weeks, presented by patient subgroup (full analysis set)

**Subgroup**	**Esomeprazole 20 mg once daily (n=130)**
Sex	
Men (n=35)	97.0% (91.1, 100)
Women (n=95)	95.5% (91.1, 99.8)
Age	
≤64 years (n=63)	96.7% (92.2, 100)
≥65 to ≤74 years (n=43)	92.3% (83.9, 100)
≥75 years (n=24)	100% (100, 100)
*Helicobacter pylori* infection	
Positive (n=57)	98.1% (94.3, 100)
Negative (n=73)	94.3% (88.8, 99.7)
Use of corticosteroids	
Yes (n=32)	96.7 (90.2, 100)
No (n=98)	95.6 (91.4, 99.8)
Type of NSAID at baseline	
COX-2-selective (n=21)^a^	94.7% (84.7, 100)
Non-selective (n=109)	96.1% (92.3, 99.8)
CYP2C19 genotype	
Poor metaboliser (n=22)	95.5% (86.8, 100)
Hetero extensive metaboliser (n=68)	93.8% (87.9, 99.7)
Homo extensive metaboliser (n=40)	100% (100, 100)

^a^Including celecoxib, etodolac and nabumetone.

**Abbreviations:** COX, cyclo-oxygenase; CYP2C19, cytochrome P450 2C19; NSAID, non-steroidal anti-inflammatory drug.

Shift tables showed that the majority of patients experienced an improvement in modified LANZA score during the course of the study (Table 5).

**Table 5:** **Number of patients by modified LANZA score**^**a **^**of the severity of gastric mucosal lesions at study end, stratified by baseline score (full analysis set)**

	**Baseline**				
**Study end**	**0**	**+1**	**+2**	**+3**	**+4**
0	40	**10**	**28**	**4**	**0**
+1	7	2	**8**	**0**	**0**
+2	5	2	11	**2**	**0**
+3	1	0	2	0	**1**
+4	1	0	3	1	2

^a^Absence of haemorrhage and erosion (0); one haemorrhage or erosion (+1); 2–10 haemorrhages or erosions (+2); 11–25 haemorrhages or erosions (+3): >25 haemorrhages, erosions or an ulcer (+4). Bold highlighted number in cell signify improvement between baseline and study end.

While there was a very low incidence of dyspeptic symptoms both at baseline and during the course of the study, the number of patients with abdominal symptoms tended to decrease during long-term treatment with esomeprazole (Table 6).

**Table 6:** Number of patients with dyspeptic symptoms at baseline and study end, stratified by baseline severity of symptoms (full analysis set)

**Symptom**		**Baseline**	
	**Study end**	**None**	**Mild–Severe**
Epigastric pain	None	100	18
	Mild–Severe	8	4
Stomach discomfort	None	102	14
	Mild–Severe	5	9
Abdominal fullness	None	100	18
	Mild–Severe	4	8
Nausea/vomiting	None	116	8
	Mild–Severe	4	2
Heartburn	None	112	14
	Mild–Severe	3	1
Anorexia	None	111	10
	Mild–Severe	7	2

## Discussion

Esomeprazole 20 mg od, administered over 1 year, had a favourable safety profile and was well tolerated in Japanese NSAID users with a history of peptic ulcer. Such findings are consistent with another recent study of treatment with lansoprazole (15 mg od) in long-term NSAID users in Japan [18]. Notably, the nature and frequency of AEs with esomeprazole in the present study was generally consistent throughout the 1-year treatment duration, and only a small proportion of patients experienced AEs that were assessed as possibly drug-related by the investigator. Moreover, few serious AEs were reported.

In terms of efficacy (secondary objective), esomeprazole proved efficacious in preventing the recurrence of peptic ulcers in this at-risk patient population, with an overall estimated ulcer-free rate after 1 year of 95.9%. Such findings compare favourably with a similar study of lansoprazole in Japanese long-term NSAID users and a history of peptic ulcer, in which the estimated ulcer-free rate after 1 year was somewhat lower at 87.3% [18]. Moreover, there was a trend for a reduction in endoscopic GI lesions (modified LANZA score) in the present study, and few patients experienced dyspeptic symptoms during study treatment. These results, together with the favourable safety and tolerability findings, are consistent with previous long-term studies of esomeprazole for prevention of pre-specified GI symptoms and peptic ulcers in European and North American patients receiving NSAIDs [12,13]. Indeed, the ulcer-free rate with esomeprazole 20 mg od in the present 1-year study (95.9%) is better than those seen in the placebo-controlled, 6-month VENUS and PLUTO studies (ulcer-free rates of 94.7% and 94.8%, respectively; life-table estimates) [13]. A double-blind, randomised placebo-controlled trial conducted in Japan also showed superior efficacy of esomeprazole in preventing NSAID-related ulcer recurrence compared with Western studies [14].

In accordance with earlier studies [13], we found that esomeprazole seemed to be similarly efficacious for prevention of peptic ulcers irrespective of the type of NSAID that the patient was being treated with (non-selective or COX-2 selective), although patient numbers were small. Unfortunately, we were not able to evaluate the efficacy of esomeprazole according to the duration of NSAID treatment prior to study start, as such information was not collected during baseline assessments. This represents a potential study limitation, as duration of prior NSAID treatment could impact on the risk of ulcer recurrence. *H. pylori* status did not seem to substantially impact the therapeutic efficacy of esomeprazole (as also observed for lansoprazole in a similar Japanese population [18]), and this is consistent with findings showing that eradication of this pathogen does not prevent recurrent ulcers in NSAID users [19]. Notably, our study population included a larger proportion of *H. pylori*-positive patients (43.8%) than comparable studies in Western populations (10–20%) [12,13].

Esomeprazole is mainly metabolised by CYP2C19 [20], which is subject to genetic polymorphism. Consequently, we performed genetic testing of CYP2C19 genotype in order to determine whether this influenced long-term efficacy of esomeprazole for prevention of peptic ulcers in our at-risk Japanese population. Overall, CYP2C19 genotype did not indicate any major impact on the gastroprotective efficacy of esomeprazole, as the estimated ulcer-free rates were similar for all CYP2C19 genotypes. While the results are limited by the small number of patients, they are in agreement with previous findings that esomeprazole-mediated healing of reflux (erosive) esophagitis is unrelated to CYP2C19 genotype [21].

We observed a low incidence of dyspeptic symptoms during the present study, which is consistent with findings elsewhere that esomeprazole provides sustained relief from such symptoms in long-term NSAID users [12]. However, it should be noted that few patients reported such symptoms at baseline; for example, only 18 patients (13.8%) reported epigastric pain with NSAID therapy. This may be somewhat discordant from studies in Western populations that have reported upper GI symptoms, such as dyspepsia, in as many as 40% of NSAID users [22]. Nevertheless, we believe our study population to be representative of Japanese NSAID users, given that the baseline rate of dyspeptic symptoms was generally comparable to the reported incidence in a recent meta-analysis of NSAID treatment studies of osteoarthritis patients in Japan [23]. This difference may be important when interpreting the dyspeptic symptom findings during long-term esomeprazole therapy.

A strength of the present study was that it used endoscopic endpoints rather than surrogate markers of peptic ulcer recurrence, such as symptom relapse. Frequent endoscopic assessment also served to ensure that the at-risk study population was closely monitored for prompt detection of ulcer recurrence. Another strength is that concomitant use of mucosal protectants such as gefarnate, which are frequently used in Japan to reduce the risk of peptic ulcers with NSAID therapy, was not permitted. This helped to ensure that the true gastroprotective effect of esomeprazole was observed, without confounding by the moderate efficacy of mucosal protection noted in comparable long-term studies [18]. The lack of a placebo or active treatment comparator could be considered a study limitation, although use of a placebo arm would be unethical in a study population at high risk of ulcer recurrence. Moreover, the existence of an extensive published literature on esomeprazole in long-term NSAID users allows some historical comparisons to be made. Indeed, findings in the present Japanese population are in agreement with results from other populations [12,13]. Another limitation is that compliance was measured by returned capsule counting and NSAID diaries. While this was the only feasible option in the current study, we have to accept the limitations of such an approach for evaluating compliance. The small size of the study also has to be considered, as this precludes the precise evaluation of rare AEs such as fracture. Indeed, there has been concern about the moderately elevated risk of fracture (hip, spine and wrist) with PPI long-term therapy based on a recent meta-analysis of observational studies [24]. However, the underlying biological mechanism(s) behind this increased risk remain unclear, and only 3 patients experienced a fracture (hand finger, rib and upper arm) in the present long-term study; in all cases the event was not considered to be related to the study drug. Concerns have also been raised about the increased risk of pneumonia with long-term PPI therapy in some [25] but not all [26] studies, although only 1 patient experienced such an event that was considered possibly-related to study drug in the present investigation. Larger studies of PPI therapy in at-risk NSAID users will be required to explore the risk of these rare AEs in greater detail.

## Conclusions

In according with previous findings, esomeprazole 20 mg od had a favourable safety profile and was efficacious at preventing ulcer recurrence during long-term treatment in Japanese NSAID users with a history of peptic ulcer. Moreover, modified LANZA scores of the severity of gastric mucosal lesions were generally improved and few patients reported dyspeptic symptoms, consistent with the gastroprotective efficacy of esomeprazole in this population.

## Competing interests

Kentaro Sugano: Advisory Board member (AstraZeneca K.K. and Takeda Pharmaceutical Co. Ltd), service honoraria (Takeda Pharmaceutical Co. Ltd) and research grants (Astellas Pharma Inc., AstraZeneca K.K., Eisai Co. Ltd, Otsuka Pharmaceutical Co. Ltd, Chugai Pharmaceutical Co. Ltd, MSD K.K. and Takeda Pharmaceutical Co. Ltd).

Yoshikazu Kinoshita: service honoraria (Astellas Pharma Inc., AstraZeneca K.K., Eisai Co. Ltd and Takeda Pharmaceutical Co. Ltd) and research grants (Astellas Pharma Inc., AstraZeneca K.K. and Eisai Co. Ltd).

Hiroto Miwa: service honoraria (Astellas Pharma Inc., AstraZeneca K.K., Eisai Co. Ltd, Dainippon Sumitomo Pharma Co. Ltd and Takeda Pharmaceutical Co. Ltd) and research grants (Astellas Pharma Inc., AstraZeneca K.K., Dainippon Sumitomo Pharma Co. Ltd and Eisai Co. Ltd and Otsuka Pharmaceutical Co. Ltd).

Tsutomu Takeuchi: service honoraria (Abbott Japan Co. Ltd, Bristol-Myers Squibb, Chugai Pharmaceutical Co. Ltd, Eisai Co. Ltd, Mitsubishi Tanabe Pharma Co. and Pfizer Japan Inc.) and research grants (Abbott Japan Co. Ltd, Astellas Pharma Inc., Bristol-Myers Squibb, Chugai Pharmaceutical Co. Ltd, Daiichi Sankyo Co. Ltd, Eisai Co. Ltd, Janssen Pharmaceutical K.K., Mitsubishi Tanabe Pharma Co., Nippon Shinyaku Co. Ltd, Novo Nordisk Pharma Ltd, Otsuka Pharmaceutical Co. Ltd, Pfizer Japan Inc., Sanofi K.K., Santen Pharmaceutical Co. Ltd, Takeda Pharmaceutical Co. Ltd and Teijin Pharma Ltd).

## Authors’ contributions

KS: study design, patient enrolment, data interpretation and manuscript preparation. YK: Central Evaluation Committee member. HM: Central Evaluation Committee member. TT: study design. All authors contributed to writing of the manuscript and approved the final version for submission.

## Pre-publication history

The pre-publication history for this paper can be accessed here:

http://www.biomedcentral.com/1471-230X/13/54/prepub
